# Emulsification Characteristics of Insoluble Dietary Fibers from Pomelo Peel: Effects of Acetylation, Enzymatic Hydrolysis, and Wet Ball Milling

**DOI:** 10.3390/foods13040624

**Published:** 2024-02-19

**Authors:** Kuimin Yang, Jieqiong Yao, Kaixin Shi, Chenxi Yang, Yang Xu, Peipei Zhang, Siyi Pan

**Affiliations:** 1 College of Food Science and Technology, Huazhong Agricultural University, Wuhan 430070, China; yangkuimin0228@163.com (K.Y.); yaojieqong@163.com (J.Y.); skx@webmail.hzau.edu.cn (K.S.); yangchenxi19990817@163.com (C.Y.); 2018309110049@webmail.hzau.edu.cn (Y.X.); zhangpeipei1217@webmail.hzau.edu.cn (P.Z.); 2 Key Laboratory of Environment Correlative Dietology, Ministry of Education, Huazhong Agricultural University, Wuhan 430070, China; 3 Hubei Key Laboratory of Fruit & Vegetable Processing & Quality Control, Huazhong Agricultural University, Wuhan 430070, China

**Keywords:** pomelo peel insoluble dietary fiber, acetylation, cellulase hydrolysis, wet ball milling, emulsifying properties

## Abstract

To improve the application potential of pomelo peel insoluble dietary fiber (PIDF) in emulsion systems, acetylation (PIDF-A), cellulase hydrolysis (PIDF-E), and wet ball milling (PIDF-M) were investigated in this paper as methods to change the emulsification properties of PIDF. The impact of the methods on PIDF composition, structure, and physicochemical properties was also assessed. The results demonstrated that both acetylation modification and cellulase hydrolysis could significantly improve the emulsification properties of PIDF. The emulsions stabilized with PIDF-A and PIDF-E could be stably stored at 25 °C for 30 d without phase separation at particle concentrations above 0.8% (*w*/*v*) and had higher storage stability: The D_4,3_ increments of PIDF-A- and PIDF-E-stabilized emulsions were 0.98 μm and 0.49 μm, respectively, at particle concentrations of 1.2% (*w*/*v*), while the storage stability of PIDF-M-stabilized emulsion (5.29 μm) significantly decreased compared with that of PIDF (4.00 μm). Moreover, PIDF-A showed the highest water retention capacity (21.84 g/g), water swelling capacity (15.40 mL/g), oil retention capacity (4.67 g/g), and zeta potential absolute (29.0 mV) among the PIDFs. In conclusion, acetylation modification was a promising method to improve the emulsifying properties of insoluble polysaccharides.

## 1. Introduction

Pickering emulsions are emulsions stabilized by solid particles adsorbed to the oil–water interface, which have important applications in the food, cosmetic, and pharmaceutical industries [1]. Food-grade polysaccharides [2] and proteins [3] possess interfacial activities, superior biocompatibility, and non-toxicity and, therefore, have become a research hotspot for replacing conventional chemical stabilizers [4]. Sustainable and biodegradable products from natural resources, such as pomelo peel [5], have gradually attracted the interests of researchers due to the currently increasing demand for natural raw materials and clean ingredients. Pomelo is a popular fruit, and the peel accounts for about 30% of its total weight. Pomelo peel has pectin, flavonoids, and active substances such as essential oils and is particularly rich in insoluble dietary fiber (IDF) [6]. Currently, pomelo peels are generally disposed as waste materials, which causes loss of their potential nutritional value and environmental problems [7]. Application of pomelo peels in emulsions can not only realize their value-added utilization but also contribute to sustainable development.

Pomelo peel insoluble dietary fiber (PIDF) has been found to have great application potential in emulsification [5] but is not an ideal emulsifier in its natural state. Its compact physical structure and the main components such as cellulose, hemicellulose, and lignin are not favorable for emulsification due to their high hydrophilicity. Introduction of hydrophobic groups such as acetyl groups can improve the amphiphilicity of polysaccharides and thus enhance their emulsifying ability [8]. Acetylation is a common and safe method to modify polysaccharides in the food industry [9,10]. It was originally used to modify starch [11] and, currently, has gained a wide range of applications in the food industry [10,12]. Recently, it has been used to modify some polysaccharides to overcome limitations caused by high hydrophilicity [10], which has attracted great interests. Acetylation modification has been reported to improve the water- and oil-holding capacity of wheat bran IDF [13]. However, there has been little research on how acetylation modification affects the structure and physicochemical properties of IDF and further influences its emulsifying properties.

Moreover, hydrolytic enzymes such as cellulase can alter the structure of polysaccharides, resulting in the exposure of more hydrophilic and hydrophobic groups to facilitate the extension of polysaccharides at the oil–water interface, thereby enhancing the emulsification effect [14,15]. Wet ball milling was reported to improve the water- and oil-holding capacity of apple pomace, and the treated apple pomace could stabilize the Pickering emulsions with good stability and antioxidant properties [16]. All these methods may affect the composition and structure of IDFs, leading to changes in the physicochemical and emulsified properties [8,17,18,19]. Nevertheless, to the best of our knowledge, there is no information about comparing the effect of acetylation, enzymatic hydrolysis, and wet ball milling treatments on the structure, physicochemical, and emulsifying properties of PIDF. Therefore, this study investigated the modification effects of acetylation, cellulase hydrolysis, and wet ball milling on PIDF to enhance its utility as a Pickering emulsifier. The findings are expected to provide important implications for the development and application of natural emulsifiers.

## 2. Materials and Methods

### 2.1. Materials

Shatian pomelos commercially available from Meizhou (Guangdong, China) were procured in their fresh and ripe state (harvesting season was November), ensuring that they were free from any pests or diseases. The fresh fruits were then meticulously cleaned and peeled, and the peels were diced into uniform small pieces. These pieces were freeze-dried, ground through an 80-mesh sieve, and preserved at 4 °C until use. Enzymes such as high-temperature-resistant α-amylase (500 U/g) and glucoamylase (1.0 × 10^5^ U/mL) were acquired from Aladdin Reagent Co. Ltd. (Shanghai, China). Additionally, papain (800 U/mg), cellulase (green xylanase, 400 U/mg), and Nile red were purchased from Shanghai Yuanye Biotechnology Co. Ltd. (Shanghai, China). Soybean oil was obtained from a Supermarket in Wuhan (Hubei, China), and acetic anhydride was sourced from Sinopharm Chemical Reagent Co., Ltd. (Shanghai, China).

### 2.2. Preparation of PIDF

A modified enzymatic method in reference to a previous study was employed to extract PIDF [7]. First, 10 g powder was dispersed in water at a material–liquid ratio of 1:20 under constant stirring with a DF-101S thermostatic magnetic stirrer (Shanghai Lichen Bangxi Instrument Technology, Co., Ltd., Shanghai, China), and a pH of 6.0 was maintained. Then, 2.5 mL of high-temperature-resistant α-amylase solution was added to the mixture. The mixture was then heated to 95 °C for 1 h using a DF-101S thermostatic magnetic stirrer, followed by rapid cooling to 60 °C and addition of 1 mL papain solution. The reaction was carried out at 60 °C for 30 min, followed by adjustment of pH to 4.5 with 3 mol/L acetic acid solution and addition of 1 mL glucoamylase solution, and the reaction was continued at 60 °C for 30 min. Subsequently, the sample solution was boiled for 15 min to deactivate the enzymes, cooled to room temperature, and centrifuged at 5590× *g* for 10 min using an LXJ-IIB centrifuge (Shanghai Anting Scientific Instrument Factory, Shanghai, China). The resulting precipitate was washed thrice with hot water at 70 °C, dried in a WGL-45B oven (Tianjin Taiste Instrument Co., Ltd., Tianjin, China) at 40 °C, crushed using a MM 400 mixer mill (Retsch GmbH, Hahn, Germany) with a stainless-steel grinding bead for 1 min at 25 Hz, sieved (80 mesh), and labeled as PIDF.

### 2.3. Modification of PIDF

#### 2.3.1. Acetylation Modification

The PIDF was subjected to acetylation modification according to the procedure described in a previous study with minor modifications [20]. The PIDF was first dispersed in deionized water under constant stirring with a DF-101S thermostatic magnetic stirrer to create a polysaccharide solution with a solid–liquid ratio of 1:15. The pH was adjusted to 8.5 using a 0.5 mol/L NaOH solution and maintained for 20 min. Subsequently, 0.5 mol/L NaOH solution and acetic anhydride were alternately added at 30 °C, ensuring that the pH was maintained between 8.0 and 10.0. Upon addition of acetic anhydride equivalent to 75% of the PIDF mass, the sample was continuously stirred for 3 h at the same temperature. The pH was adjusted to 7.0 using a 0.5 mol/L HCl solution to terminate the reaction. The reaction mixture was then concentrated to 1/4 of the original volume using a rotary evaporator (R–1001VN, Greatwall, Zhengzhou, China) under vacuum at 0.08–0.095 mPa and at 50 °C. Anhydrous ethanol at four times volume of the concentrated mixture was added for overnight precipitation at 4 °C. The acetylation-modified PIDF (PIDF-A) was then dried in a WGL-45B oven at 40 °C, crushed using a MM 400 mixer mill with a stainless-steel grinding bead for 1 min at 25 Hz and sieved (80 mesh).

The degree of acetylation substitution (DS) for PIDF-A was 0.12 as determined following the same procedure described by Huang et al. [10], and PIDF was used as the control. Briefly, 20 mg of PIDF-A was dispersed in 10 mL of 0.01 mol/L NaOH solution. The mixture was then heated to 50 °C for 2 h at 150 rpm with a water bath constant temperature oscillator (DSHZ-300A, Suzhou Peiying Experimental Equipment Co., Suzhou, China) and then cooled to room temperature, and the excess NaOH was titrated with 0.01 mol/L HCl solution, with phenolphthalein as an indicator. The DS was calculated according to the following equations:

A (%) = [(V_PIDF_ − V_PIDF-A_) × C × 0.043 × 100]/W (1)

DS (%) = (162 × A%)/(4300 − 42 × A%)
(2)

where A is the acetyl group content, V_PIDF_ is the volume of HCl required for blank (PIDF), V_PIDF-A_ is the volume of HCl required for PIDF-A sample, C is the concentration of HCl (mol/L), and W is the mass of sample (g).

#### 2.3.2. Enzymatic Modification

The enzymatic modification of PIDF was carried out according to the method of Gao et al. with slight modifications [15]. Specifically, 30 g of PIDF powder was suspended in water with a solid–liquid ratio of 1:25 and pH of 4.5. Then, 0.625 g cellulase was added, and the sample was stirred at 50 °C for 4 h using a DF-101S thermostatic magnetic stirrer. The suspension was then heated to 100 °C for 15 min to deactivate the enzymes, cooled to room temperature, and precipitated overnight using anhydrous ethanol. Finally, it was dried in a WGL-45B oven at 40 °C, crushed using a MM 400 mixer mill with a stainless-steel grinding bead for 1 min at 25 Hz, and then sieved (80 mesh) to obtain enzymatic-hydrolysis-modified PIDF (PIDF-E).

#### 2.3.3. Modification by Wet Ball Milling

A wet ball mill (MiniZeta 03E, Netzsch (Shanghai) Machinery & Instruments Co., Ltd. (Shanghai, China)) was used to modify the PIDF by referring to the method of Lu et al. with appropriate adjustments [16]. Zeta beads (0.4–0.6 mm) served as the grinding medium, and deionized water was used as the solvent. The sample-to-solvent mass ratio was set at 1: 25, with a rotational speed of 3000 r/min and a grinding power of 750 W. The ball milling process was sustained for 8 h. Finally, the ball-milled samples were sealed and stored at 4 °C for future experiments, and a portion of samples were precipitated with anhydrous ethanol overnight and dried in a WGL-45B oven at 40 °C, crushed using a MM 400 mixer mill with a stainless-steel grinding bead for 1 min at 25 Hz, and then sieved (80 mesh) to produce wet-ball-milled modified PIDF (PIDF-M) for subsequent analysis.

### 2.4. PIDF Chemical Composition and Physicochemical Properties

#### 2.4.1. Chemical Composition Determination

The contents of cellulose, hemicellulose, and lignin were quantified using a Ringbio fully automated fiber analyzer (R-2000, Ringbio Instruments, London, UK) [21]. Briefly, the samples of PIDFs were treated sequentially with neutral detergent, acid detergent, 72% H_2_SO_4_, and high-temperature scorching in a muffle furnace (SX-4-10, Changzhou Ronghua Instrument Manufacturing Co., Ltd., Changzhou, China). Then, the neutral detergent fiber (NDF), acid detergent fiber (ADF), acid detergent lignin (ADL), and ash content were measured. Finally, the cellulose, hemicellulose, and lignin contents were calculated based on the difference between ADF and ADL, the difference between NDF and ADF, and the value of ADL, respectively [22]. The protein content was determined using a Kjeldahl nitrogen meter (KDN-04A, MINCEE, Xiamen, China) according to the Kjeldahl method with a conversion factor of 6.25. The fat content was determined via the Soxhlet extraction method using a fat analyzer (SOX406, Hanon Instruments Co., Ltd., Jinan, China). The moisture content was quantified by direct drying method using a WGL-45B oven to allow the samples to reach constant weight.

For monosaccharide composition analysis, 10 mg of the sample was subjected to hydrolysis in a tube with 5 mL of 2 mol/L trifluoroacetic acid under an N₂ atmosphere at 110 °C for 4 h in an oil bath. After hydrolysis, the trifluoroacetic acid was eliminated through spin-drying (R–1001VN, Greatwall, Zhengzhou, China), and the residue was washed thrice using 2 mL of methanol. The resultant product was re-dissolved in 2 mL of water. A 500 μL aliquot of this solution was subsequently mixed with 500 μL 0.25 mol/L NaOH and 500 μL PMP (1-phenyl-3-methyl-5-pyrazolone) methanol solution and allowed to react for 90 min at 70 °C in a water bath (HH-2, Guohua, Changzhou, China). Subsequently, the solution was cooled to room temperature and the pH was neutralized by 0.3 mol/L HCl solution, followed by extraction with 1 mL of trichloromethane and filtration. High-performance liquid chromatography (HPLC, Ultimate 3000, Thermo Fisher Scientific, Waltham, MA, USA) was employed for the analysis, with linear regression analysis to correct the relationship between monosaccharide concentration and peak area. The HPLC conditions were set as follows: a ZORBAX Eclipse XDB-C18 column (4.6 mm × 250 mm, 5 μm); a mobile phase comprising 0.1 mol/L phosphate buffer (pH 6.7) and acetonitrile in an 83: 17 *v*/*v* ratio; a column temperature of 30 °C; a detection wavelength of 250 nm; a flow rate of 1 mL/min; and an injection volume of 10 μL.

#### 2.4.2. Particle Size Determination

The particle size of the sample was measured using an APA2000 laser particle size analyzer (Malvern Mastersizer, Malvern, UK) with the refractive index of deionized water set at 1.33. Specifically, a 2% (*w*/*v*) PIDF aqueous solution was prepared and added to the dispersion unit connected to the laser particle size analyzer until the degree of masking was approximately 10%. The particle size data were expressed as the volume-weighted average particle diameter D_4,3_.

#### 2.4.3. Fourier-Transform Infrared Spectroscopy (FT-IR)

FT-IR using a Nicolet IS50 spectrometer (Thermo Fisher Scientific, Waltham, MA, USA) was employed to characterize the functional groups in the samples. The sample powder was homogeneously mixed with KBr powder in a 1:100 *v*/*v* ratio and pressed into transparent sheets for analysis, covering a spectral frequency range of 500–4000 cm⁻^1^.

#### 2.4.4. X-ray Diffraction (XRD)

The crystalline structure of PIDF samples was characterized using an X-ray diffractometer (D8 Advance, Bruker, Karlsruhe, Germany) operated at 40 kV and 40 mA. The diffraction angle (2θ) was scanned from 5° to 55° in 0.02° increments at a rate of 6°/min. The acquired diffracted intensity data were utilized to calculate the crystallinity of the PIDF samples according to the Segal method [23].

#### 2.4.5. Scanning Electron Microscopy (SEM)

The surface morphology of the samples was investigated using a JSM-6390LV scanning electron microscope (JEOL, Tokyo, Japan). Samples were mounted on brass discs using double-sided conductive carbon tape and coated with gold by ion sputtering. Observations were conducted at 1000× and 3000× magnifications under an accelerating voltage of 10 kV.

#### 2.4.6. Water Retention Capacity (WRC)

The WRC of samples was calculated according to the method described by Zhang et al. with some modifications [24]. A 0.10 g sample was mixed with 10 mL of deionized water for 24 h at room temperature, centrifuged (Allegra X-30R, Beckman Coulter, Brea, CA, USA) at 2632× *g* for 15 min, and the supernatant was removed by slowly tilting the centrifuge tube. The residue was then weighed to determine the WRC using the formula:

WRC (g/g) = (m_2_ − m_0_)/(m_1_ − m_0_) (3)

where m_0_ is the mass of the empty centrifuge tube, m_1_ is the total mass before water absorption, and m_2_ is the total mass after water removal.

#### 2.4.7. Water Swelling Capacity (WSC)

The WSC of samples was determined according to the method described by Wang et al. with minor adjustments [25]. A 0.20 g sample was placed in a 10 mL graduated test tube, and the volume was recorded, followed by the addition of 5 mL of deionized water. After resting at 4 °C for 18 h, the post-absorption volume of the sample was recorded, and the WSC was calculated as follows:

WSC (mL/g) = (V_1_ − V_0_)/m (4)

where V_1_ is the final volume, V_0_ is the initial volume, and m is the mass of the sample.

#### 2.4.8. Oil Retention Capacity (ORC)

The ORC of samples was determined following the procedure described by Zhang et al. with some modifications [24]. A 0.10 g sample was mixed with 10 mL of soybean oil for 1 h, centrifuged at 5000 r/min for 15 min, and the supernatant was discarded. The residue was blotted to remove excess oil and then weighed to calculate the ORC as follows:

ORC (g/g) = (M_2_ − M_1_)/M_1_ (5)

where M_2_ is the wet weight and M_1_ is the dry weight of the sample.

#### 2.4.9. Zeta Potential Measurement

The zeta potential of a 0.1% (*w*/*v*) aqueous solution of the sample was assessed using a Nano ZS nanoparticle size and potential analyzer (Malvern Zetasizer, Malvern, UK).

### 2.5. Emulsion Preparation and Property Characterization

#### 2.5.1. Preparation and Macroscopic Evaluation of Emulsions

According to the preliminary test results, the O/W emulsion was prepared by PIDF, PIDF-A, PIDF-E, and PIDF-M at different particle concentrations with an oil–water ratio of 2:8, in which the particle concentrations were 0.4%, 0.8%, 1.2%, 1.6%, and 2% (*w*/*v*), respectively, which were expressed as weight content relative to the aqueous phase. A high shear homogenizer (FJ300-SH, Biaoben Instruments, Shanghai, China) was used to mix the emulsifier, deionized water, and soybean oil at 10,000 rpm for 2 min. Then, a high-pressure homogenizer (AH-2010, ATS Engineering Inc., Brampton, ON, Canada) was used to circulate for three times at 750 bar to obtain a fine emulsion. The volumetric appearance of the emulsion prepared at different concentrations after 24 h and 30 d of storage at 25 °C was recorded with a mobile phone camera (Find X2, Oppo, Shenzhen, China).

#### 2.5.2. Droplet Size Analysis of Emulsions

The mean droplet size of the emulsions, both freshly prepared and after storage, was determined with a laser particle size analyzer (APA2000, Malvern Mastersizer, Malvern, UK). The relative refractive index of the emulsion was determined by dividing the refractive index of the oil (1.456) by the refractive index of the continuous phase (1.33), which yielded a value of 1.095 [5]. A homogeneous emulsion sample was added to the dispersion unit connected to the laser particle size analyzer until the degree of masking was approximately 10%. The particle size data were expressed as the volume-weighted average particle diameter D_4,3_.

#### 2.5.3. Confocal Laser Scanning Microscopy (CLSM)

Following the method of Huang et al., the microstructure of the emulsion was observed using CLSM (FV3000, Olympus, Tokyo, Japan) with minor modifications [10]. About 20 μL of a 1% (*w*/*v*) Nile Red dye was added to 1 mL of emulsion sample and mixed well. The stained emulsion sample was then gently dropped onto the glass slide and covered with a coverslip to ensure that the sample was flat. The excitation wavelength of Nile Red was set to 561 nm, and a 60× objective was used to obtain a clear image of the microstructure.

#### 2.5.4. Rheological Behavior Assessment

The rheological properties of fresh emulsions were determined using a rheometer (DHR2, TA Instruments, New Castle, DE, USA) at 25 °C. The diameter of the parallel plate used was 40 mm, and the gap was set at 1000 µm. In steady-state shear scanning, the apparent viscosity of the emulsion as a function of the shear rate (ranging from 10 to 1000 s⁻^1^) was studied. Before the dynamic frequency sweep, strain sweep (0.01–100%) tests were performed to assess the linear viscoelastic region. In the dynamic sweep test, the values of elastic modulus (G′) and the loss modulus (G″) were recorded, and the frequency variation range was set to 0.1–10 rad/s. All measurements were made in the linear viscoelastic region and performed at a fixed strain of 1%.

### 2.6. Statistical Analysis

All experiments were conducted in at least triplicate. Data were statistically analyzed using SPSS software (version 26.0, IBM Corp., Armonk, NY, USA), and the results of the experiments were subjected to one-way ANOVA using Duncan’s test. Significant differences were determined to be statistically significant at *p* < 0.05.

## 3. Results and Discussion

### 3.1. Chemical Composition and Particle Size Analysis

The effects of the three modification methods on the chemical composition of PIDF and its particle size are presented in Table 1. In general, these modification methods did not significantly affect the fat and ash content of PIDF (*p* > 0.05) but decreased the protein, cellulose, hemicellulose, and lignin contents (*p* < 0.05), indicating partial degradation of PIDF during the modification process.

Monosaccharide composition analysis revealed that both PIDF and modified PIDFs were composed of eight monosaccharides. Glucose and arabinose were predominant monosaccharides in PIDF, followed by galactose, xylose, mannose, and rhamnose, whereas galacturonic and glucuronic acids were rare monosaccharides. The abundance of glucose can be attributed to the high cellulose content in PIDF [26]. Compared with PIDF, PIDF-A exhibited a lower proportion of glucose while higher proportions of other monosaccharides, particularly arabinose and galactose, which is consistent with the results in a previous study [10]. In another study [27], acetylation treatment was found to lead to possible degradation of polysaccharides. These changes in monosaccharide profile may be ascribed to glycosidic bond cleavage during modification, which produces various free monosaccharides, disaccharides, or oligosaccharides [10], thereby influencing the polysaccharide composition in the final products. Compared with PIDF, PIDF-E showed a notable reduction in glucose content, which agrees with the findings in citrus fibers [26], and increases in arabinose and galactose contents, probably due to the conversion of cellulose and hemicellulose to soluble dietary fiber (SDF) by cellulase. The increase in xylose content is associated with a relative increase in hemicellulose content [28]. PIDF-M showed reduction in galacturonic acid content, and Song et al. also found a similar result that the galacturonic acid content of citrus fiber decreased after ball milling modification [26]. The content of galacturonic acid is mainly found in pectin [29]. The decrease in galacturonic acid content after PIDF-M treatment compared to PIDF may be attributed to the numerous changes in the structure of PIDF-M during ball milling, which causes pectin attached to cellulose or hemicellulose chains to fall off and be removed by ethanol in the subsequent process [26,30]. 

Particle size analysis revealed the particle size of PIDF-A, PIDF-E, and PIDF-M significantly decreased to 78.17 ± 0.69, 66.13 ± 1.66, and 57.63 ± 3.91 μm, respectively, relative to the 145.21 ± 3.33 μm of PIDF (Table 1, Figure S1). The particle size reduction in PIDF-A may be attributed to the partial destruction of the structure of PIDF by acetylation [11], that in PIDF-E may be due to the disruption of fibrous chains by hydrolysis [31], while that in PIDF-M may be ascribed to the mechanical action of ball milling that causes large particles to be broken down into smaller ones [16].

### 3.2. FT-IR Analysis

**Figure 1:**
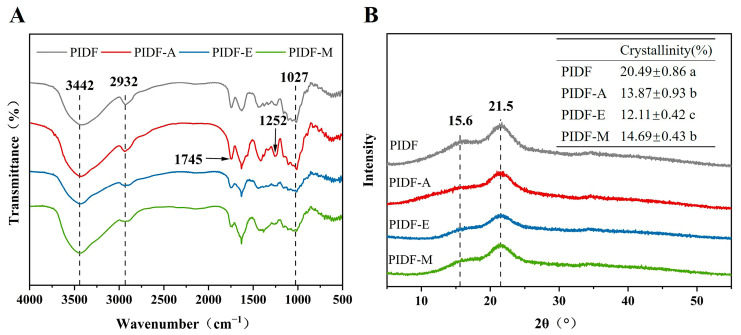
(**A**) Fourier-transform infrared spectroscopy and (**B**) X-ray diffraction of PIDF, PIDF-A, PIDF-E, and PIDF-M. Different lowercase letters (a–c) in the table in subfigure B indicate significant differences (*p* < 0.05).

FT-IR was employed to identify the functional groups across diverse PIDF samples (Figure 1A). Interestingly, all samples exhibited characteristic FT-IR spectra of polysaccharides [32], despite minor deviations in absorbance and wavenumber. The peaks at 3442, 2932, and 1027 cm⁻^1^ can be attributed to hydroxyl O-H stretching, C-H stretching in methyl, methylene, and methine groups, and C-O stretching vibration, respectively [31,33,34]. Specifically, the absorption peak of PIDF-A at 3442 cm⁻^1^ significantly increased, probably because more free hydroxyl groups were exposed and replaced by acetyl groups, which is consistent with the results of other studies of acetylated polysaccharides [31]. Moreover, the incorporation of acetyl groups was further confirmed by the enhanced absorption peaks of PIDF-A at 1745 cm⁻^1^ and 1252 cm⁻^1^, which correspond to the carbonyl C=O vibration of the acetyl group and C-O tensile vibration of the carbonyl group, respectively [10]. Conversely, the peak intensity of PIDF-E at 3442 cm⁻^1^ and 2932 cm⁻^1^ decreased compared with that of PIDF, probably due to disruption of partial hydrogen bonds as well as methyl and methylene components in hydrogen bonds [35]. 

### 3.3. XRD Analysis

The XRD of PIDF, PIDF-A, PIDF-E, and PIDF-M is shown in Figure 1B. All samples showed diffraction peaks at around 15.6° and 21.5° and exhibited no significant difference in peak position, indicating that the three modification methods do not significantly affect the crystal structure of cellulose in PIDF. The crystallinity of PIDF was 20.49%, while PIDF-A, PIDF-E, and PIDF-M showed 32.31%, 40.90%, and 28.31% decreases in crystallinity relative to that of PIDF, respectively. These decreases may be attributed to the disruption of partial intramolecular hydrogen bonds, which serve as the main source of stability in cellulose crystal regions [36]. It should be noted that a decrease in crystallinity means a decrease in crystalline regions and accordingly an increase in amorphous regions. It has been reported that most reactive reagents can more easily penetrate into the amorphous region than into the dense crystalline region of cellulose [37]. Therefore, it can be speculated that the changes in physicochemical properties of the modified PIDF may be related to decreases in crystallinity. In addition, the decrease in crystallinity may improve the WRC and WSC of IDF as reported by Zheng et al. [18].

### 3.4. SEM Analysis

**Figure 2:**
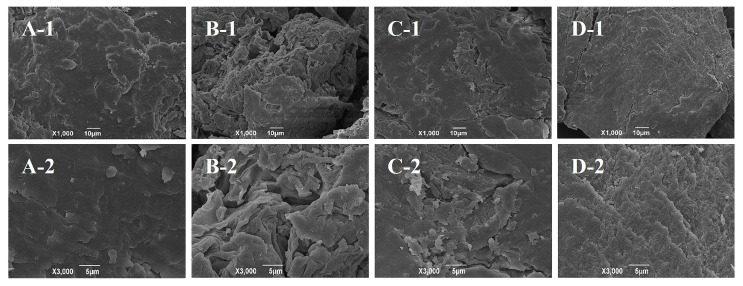
Scanning electron micrographs of PIDF at 1000× and 3000× magnifications: PIDF (**A-1**, **A-2**), PIDF-A (**B-1**, **B-2**), PIDF-E (**C-1**, **C-2**), and PIDF-M (**D-1**, **D-2**).

Surface microstructure has a significant impact on the functional properties of PIDF. Specifically, a richer fold structure and deeper porosity depth are expected to improve the emulsifying activity and emulsion stability of PIDF [38]. As shown in Figure 2, modification changed the microstructure of PIDF to varying degrees. PIDF exhibited a relatively tight microstructure (Figure 2A), but PIDF-A showed more wrinkles and a porous surface (Figure 2B). This change is similar to that reported in the literature [11,12] and likely due to the destruction of the surface micromorphology by esterification to some extent [12]. The surface of PIDF-E showed many obvious cracks, accompanied by the formation of numerous small fragments or particles (Figure 2C). These changes led to higher porosity and surface area of PIDF-E, which may be due to the degradation of wall polysaccharides by cellulase hydrolysis [39]. PIDF-M exhibited a rougher, uneven, and tighter surface structure with shallower pores and lower porosity (Figure 2D), which is most likely due to the effect of mechanical force of wet ball milling. In general, various modification methods could change the surface microstructure of PIDF to varying degrees, and acetylation and enzymatic hydrolysis may have a positive impact on the functional properties of PIDF. 

### 3.5. Hydration Properties Analysis

**Figure 3:**
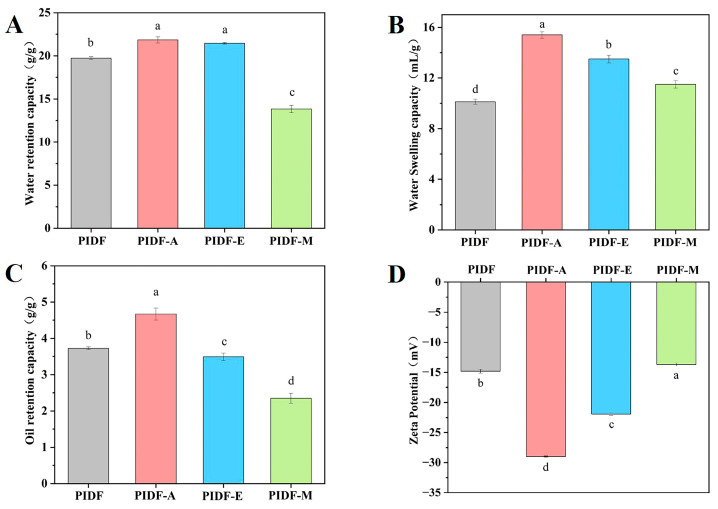
Physicochemical properties of modified and non-modified PIDF: WRC (**A**), WSC (**B**), ORC (**C**), and zeta potential (**D**). Different lowercase letters (a–d) indicate significant differences (*p* < 0.05).

Figure 3 reveals obvious differences in the physicochemical properties of PIDF before and after modification. Compared with that of PIDF (19.73 g/g), the WRC of PIDF-A and PIDF-E (Figure 3A) increased by 10.69% and 8.72%, respectively. Conversely, the WRC of PIDF-M decreased by 29.95%. The increase in WRC by acetylation may be due to the exposure of more free hydroxyl groups in PIDF and formation of a porous structure in an alkaline environment, which is strongly supported by the FT-IR and SEM analysis results. Cellulase hydrolysis can increase the content of SDF by breaking down hemicellulose and cellulose [28,37]. Moreover, it has been reported that WRC is positively correlated with the SDF content [35]. These facts can explain the higher WRC of PIDF-E. Wet ball milling reduces the surface porosity of PIDF, which in turn reduces its ability to bind water and therefore results in lower WRC of PIDF-M.

Figure 3B shows that the three different modification strategies significantly improved the WSC of PIDF (*p* < 0.05). PIDF-A exhibited the highest WSC, which may be related to its pleated and porous surface structure. Cellulase hydrolysis exposes more hydrogen bonds and water-binding sites by breaking the β-glycosidic bonds, thereby resulting in higher WSC of PIDF-E [35]. Moreover, PIDF-M also exhibited a higher WSC, probably due to its smaller particle size, which improves water absorption and swelling [40].

As shown in Figure 3C, PIDF-A had the most significant ORC, which was enhanced by 25.2% relative to that of PIDF. This enhancement can be attributed to two reasons. First, the porous surface structure and higher specific surface area may increase the contact of the fibers with oil/water molecules, thus improving the ORC/WRC [35,41], which is consistent with the results of PIDF-A in Figure 2 and Figure 3A,C; and second, hydrophobic groups are introduced into PIDF molecules under the action of acetylation, which contributes to the improvement of the ORC of PIDF. However, PIDF-E showed decreases in ORC (3.49 g/g, *p* < 0.05) relative to PIDF (3.73 g/g), which is consistent with the finding of Zheng et al. [18] that enzymatic treatment tends to reduce ORC. The decrease in ORC of PIDF-M may be related to a decrease in its surface porosity and the corresponding decrease in its ability to bind with oil.

### 3.6. Zeta Potential Analysis

Electrostatic repulsive force plays a key role in inhibiting the coalescence of suspended particles [5]. In general, a higher zeta potential absolute value represents a more stable system [38], because an increase in the number of charges in the dispersed system will strengthen the intermolecular electrostatic repulsive force and further inhibit particle coalescence [26]. As shown in Figure 3D, PIDF-A and PIDF-E demonstrated significantly higher absolute values of zeta potential than PIDF (*p* < 0.05), suggesting that they have higher solution stability. In particular, PIDF-A displayed the highest absolute value of zeta potential, indicating that the acetyl group is successfully introduced into PIDF molecules through the reaction between the -OH group in PIDF and the acetyl group in acetic anhydride. Acetylation treatment not only increased the charge but also introduced hydrophobic functional groups, which further optimized the properties of PIDF. It has been reported that higher hydrophobicity helps to maintain the stability of oil–water interface, while a high absolute value of zeta potential increases the electrostatic repulsion between droplets [5]. Notably, PIDF-E also had significantly higher absolute values of zeta potential than PIDF, suggesting that cellulase hydrolysis can disintegrate the internal structure of PIDF, resulting in the exposure of more negatively charged groups [5]. However, PIDF-M showed a decrease in absolute zeta potential value, which is not in agreement with some previous findings that wet ball milling increases the zeta potential magnitude of soybean residue [40]. This discrepancy may be caused by multiple factors such as sample characteristics or milling parameters.

### 3.7. Properties of O/W Emulsions Stabilized by Different PIDFs

#### 3.7.1. Emulsion Appearance and Particle Size Analysis

**Figure 4:**
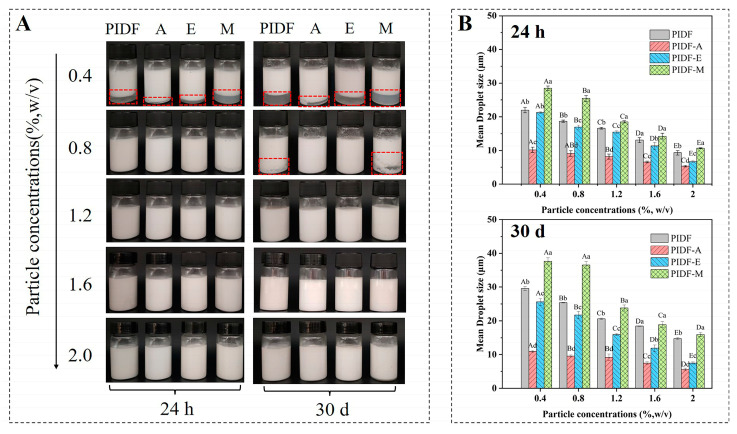
Appearance (**A**) and droplet size (**B**) of stabilized emulsions of different PIDFs: storage at 25 °C for 24 h and 30 d. The red frames in figure A indicate emulsion phase separation. Different uppercase letters indicate statistically significant differences among different particle concentrations of each sample (*p* < 0.05), and different lowercase letters indicate statistically significant differences among different samples at the same particle concentration (*p* < 0.05).

As shown in Figure 4A, the appearance of emulsions showed significant differences after 24 h and 30 d of storage at 25 °C. At the particle concentration of 0.4% (*w*/*v*), all emulsions showed poor stability and different degrees of phase separation. However, the stability of emulsions was significantly improved with increasing particle concentration, probably because more particles are transported to the oil droplets after a moderate increase in particle concentration [16]. Compared with other types of emulsions, PIDF-A and PIDF-E emulsions exhibited higher stability. In particular, at a particle concentration of 0.8% (*w*/*v*), neither emulsion exhibited the phenomenon of phase separation, which may be closely related to their high surface charge.

The droplet sizes of different PIDF-stabilized emulsions are shown in Figure 4B. It can be observed that the droplet size decreased with increasing particle concentration, which is in accordance with the general tendency of O/W emulsions stabilized by solid particles [16]. The emulsion droplet size stabilized by PIDF-A was the smallest at the same particle concentration, which further verified that acetylation can effectively enhance the emulsification performance of PIDF [38], probably because the grafting of acetyl groups increases the amphiphilicity of IDF and reduces the interfacial tension, thereby enabling its rapid adsorption to the oil–water interface [10].

#### 3.7.2. Storage Stability of Emulsions

Average droplet size and appearance of the emulsion are important indicators for the storage stability of emulsions [16]. After 30 d storage at an ambient temperature of 25 °C, the emulsions showed visually discernible changes. A number of samples presented slight phase separation, forming a biphasic system (Figure 4A, highlighted in red). Notably, PIDF-M emulsions exhibited the most pronounced phase separation of emulsion layers.

Previous studies have demonstrated a direct negative correlation between the stability of an emulsion and its droplet size [38]. In order to quantitatively assess the storage stability of the emulsions, we compared the D_4,3_ values of the emulsion samples after 24 h and 30 d of storage (Figure 4B). The D_4,3_ of PIDF, PIDF-A, PIDF-E, and PIDF-M was significantly increased by 4.00 ± 0.06, 0.98 ± 0.22, 0.49 ± 0.22, and 5.29 ± 0.74 μm (Figure S2D), respectively, after 30 d of storage at 1.2% (*w*/*v*) particle concentration. The variation in their droplet size distributions is shown in Figure S2C. The droplet size distributions of the emulsions stabilized by PIDF-A and PIDF-E remained more stable, indicating that PIDF-A and PIDF-E showed superior performance in inhibiting droplet size growth. The increased storage stability of PIDF-A- and PIDF-E-stabilized emulsions may be attributed to various factors such as the Pickering effect, hydrophilicity and lipophilicity nature of the samples, rheological properties (viscosity), and mutual repulsion effect between droplets. Previous studies have found that cellulase-modified pomelo peel dietary fiber can be used as a good emulsifier, and the mechanism is mainly attributed to the combination of the Pickering effect and three-dimensional network [15]; acetylation-modified polysaccharides exhibit high emulsion stability, which is attributed to their high viscosity and thick interfacial layer providing strong steric hindrance [10]. Furthermore, the storage stability experiments indicated that the minimum particle concentration of the emulsifier required to keep the emulsions stable was 1.2% (*w*/*v*). Considering the economic cost for practical industrial applications, emulsions with an emulsifier content of 1.2% (*w*/*v*) were used for the subsequent experiments to further investigate the performance of the PIDFs-stabilized emulsions. 

#### 3.7.3. Microstructure Analysis

**Figure 5:**
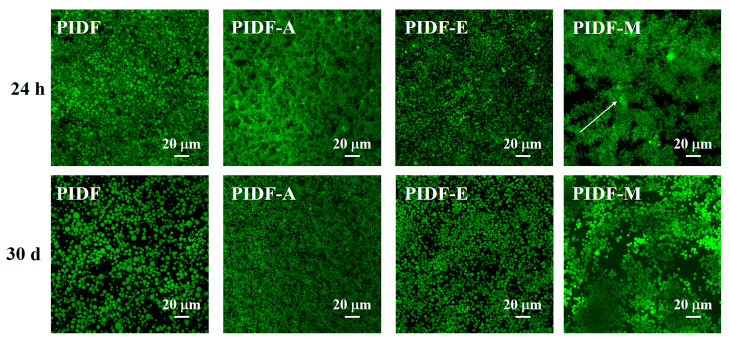
CLSM images of emulsions stabilized with 1.2% (*w*/*v*) emulsifiers stored at 25 °C for 24 h and 30 d.

At an elevated 1.2% (*w*/*v*) particle concentration, the visual differentiation among emulsions became unclear. Therefore, we further investigated the microstructure of the emulsions using CLSM technology (Figure 5) to evaluate the differences in emulsifying properties between PIDF and modified PIDFs. It is noteworthy that the droplet sizes presented by the microscopy image analysis were smaller than those obtained by the laser particle size analyzer (Figure 4B), which may be attributed to the sensitivity of the laser particle size analyzer to large droplets (Figure S2) [16,42]. The results in Figure 5 showed that PIDF-stabilized emulsions exhibited heterogeneous droplet dimensions and distribution. In contrast, PIDF-A and PIDF-E exhibited superior performance, particularly PIDF-A, which displays both smaller droplet size and more uniform distribution. Conversely, PIDF-M-stabilized emulsions were markedly unstable and are characterized by pronounced droplet flocculation and aggregation. This destabilization is likely caused by the structural disruption of PIDF polysaccharides during wet ball milling, which consequently reduces the surface porosity and the absolute zeta potential value, thereby compromising the emulsion stability. In summary, both acetylation and enzymatic treatments can markedly enhance the emulsifying performance of PIDF.

#### 3.7.4. Rheological Properties

**Figure 6:**
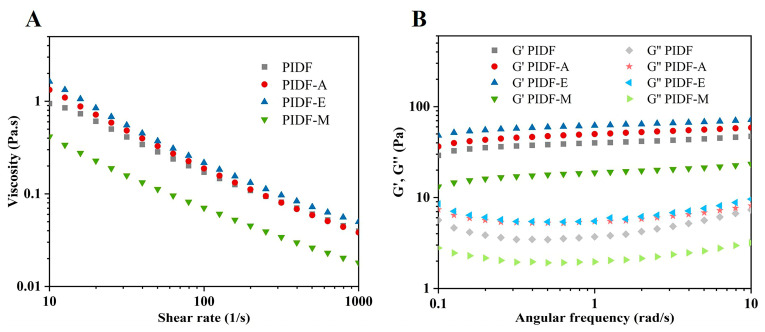
Rheological properties of freshly prepared emulsions stabilized with 1.2% (*w*/*v*) of emulsifiers: (**A**) apparent viscosity dependence on shear rate and (**B**) G′ and G″ dependence of angular frequency measured at a strain of 1.0%.

Figure 6A shows that the rheological profiles of various types of emulsions stabilized by 1.2% (*w*/*v*) PIDFs were consistent at different shear rates: The apparent viscosity decreases gradually with increasing shear rate, showing shear thinning characteristics. These results suggest that emulsions stabilized by PIDFs belong to non-Newtonian fluids [43]. Among the modified PIDFs, PIDF-A and PIDF-E showed relatively higher viscosities, which is in accordance with the viscosity change of PIDFs suspension (Figure S3). The lowest viscosity was found in the emulsion stabilized by PIDF-M, which might be one of the reasons for its relatively poor emulsion stability. Huang et al. pointed out that the combination of low viscosity and low electrostatic repulsion tends to increase the droplet size, which is not conducive to the stability of emulsions [10].

Further observation of the viscoelastic behavior of all emulsions (Figure 6B) revealed that the elastic modulus (G′) is higher than the viscous modulus (G″) over the entire frequency range, implying that these emulsions mainly exhibit elastic-dominant behavior. In this system, the fiber particles act as “scaffolds” between oil droplets to construct a robust three-dimensional network. The values of G′ and G″ increased slightly with increasing scanning frequency, indicating that the emulsions have more solid-like viscoelastic properties [43]. Among the PIDF-stabilized emulsions, the modulus magnitude relationship was PIDF-E > PIDF-A > PIDF > PIDF-M, implying that intramolecular entanglement and interchain interaction were enhanced in PIDF-A and PIDF-E [5]. This phenomenon can be attributed to the reduction in droplet size after modification, which leads to a substantial increase in the number of droplets per unit volume and in turn enhances the contact and interactions between droplets [10]. Overall, both high viscosity and small droplet size are key factors contributing to emulsion stability [27], which is consistent with the improved emulsion stability of PIDF-A and PIDF-E.

## 4. Conclusions

This study showed that acetylation and cellulase hydrolysis modification significantly improve the emulsifying performance of PIDF. Emulsions stabilized with PIDF-A and PIDF-E could be stably stored at 25 °C for 30 d without phase separation at particle concentrations above 0.8% (*w*/*v*). In particular, PIDF-A not only stabilizes more oil–water interfaces but also forms smaller and more stable emulsion droplets. In terms of physical and chemical properties, PIDF-A and PIDF-E could effectively increase the absolute zeta potential value, WRC, and WSC, among which PIDF-A showed the highest WRC (21.84 g/g), WSC (15.40 mL/g), ORC (4.67 g/g), and zeta potential absolute (29.0 mV). PIDF-E-stabilized emulsion had the highest viscosity, but PIDF-E had lower ORC than PIDF (*p* < 0.05). However, wet ball milling treatment only increased the WSC (11.50 mL/g) of PIDF. In conclusion, both acetylation and cellulose hydrolysis modification are effective strategies to improve the physicochemical properties and emulsification properties of PIDF, and PIDF-A has great potential to be used as a fat substitute and emulsifier in the food industry due to its high hydrophobicity and abundant hydrophilic groups. However, the safety of these modified PIDFs and their performance in practical application still need to be further explored in future studies.

## Figures and Tables

**Table 1:** Basic chemical composition and particle size of PIDF and the modified products.

	PIDF	PIDF-A	PIDF-E	PIDF-M
Cellulose (%)	60.05 ± 0.98 a	46.32 ± 1.06 c	38.41 ± 1.14 d	50.49 ± 0.89 b
Hemicellulose (%)	18.43 ± 0.67 a	14.59 ± 0.36 c	15.80 ± 0.69 b	13.72 ± 0.64 c
Lignin (%)	8.58 ± 0.83 a	6.82 ± 0.31 b	6.48 ± 0.23 b	7.44 ± 0.35 b
Mannose (%)	4.78 ± 0.08 b	4.91 ± 0.05 b	5.65 ± 0.06 a	4.52 ± 0.06 c
Rhamnose (%)	2.31 ± 0.04 c	2.27 ± 0.07 c	3.74 ± 0.07 a	3.59 ± 0.05 b
Glucuronic acid (%)	0.54 ± 0.06 a	0.59 ± 0.04 a	0.57 ± 0.05 a	0.57 ± 0.03 a
Galacturonic acid (%)	0.98 ± 0.04 a	1.02 ± 0.06 a	0.98 ± 0.06 a	0.70 ± 0.03 b
Glucose (%)	49.57 ± 0.42 a	35.66 ± 0.33 c	30.83 ± 0.09 d	41.09 ± 0.19 b
Galactose (%)	10.10 ± 0.17 d	15.19 ± 0.19 a	13.72 ± 0.04 b	12.17 ± 0.07 c
Xylose (%)	8.72 ± 0.08 d	9.14 ± 0.07 c	14.17 ± 0.11 a	11.71 ± 0.06 b
Arabinose (%)	23.00 ± 0.32 d	31.22 ± 0.18 a	30.33 ± 0.13 b	25.65 ± 0.06 c
Moisture (%)	3.24 ± 0.22 a	3.30 ± 0.16 a	3.31 ± 0.10 a	3.27 ± 0.16 a
Ash (%)	2.36 ± 0.28 a	2.40 ± 0.13 a	2.29 ± 0.15 a	2.51 ± 0.18 a
Protein (%)	2.65 ± 0.13 a	1.85 ± 0.14 c	2.39 ± 0.11 b	1.34 ± 0.06 d
Fat (%)	0.63 ± 0.05 a	0.60 ± 0.04 a	0.54 ± 0.11 a	0.57 ± 0.14 a
D_4,3_ (μm)	145.21 ± 3.33 a	78.17 ± 0.69 b	66.13 ± 1.66 c	57.63 ± 3.91 d

Note: Values marked with different letters in the same row indicate statistically significant differences (*p* < 0.05).

## Data Availability

The original contributions presented in the study are included in the article, further inquiries can be directed to the corresponding author.

## Supplementary Materials

The following supporting information can be downloaded at https://www.mdpi.com/article/10.3390/foods13040624/s1, Figure S1: Particle size distribution of PIDF and the modified products. Different lowercase letters (a–c) indicate significant differences (*p* < 0.05). Figure S2: (A–C) Droplet size distributions of emulsions stabilized with 1.2% (*w*/*v*) emulsifiers, (D) Droplet size increment of emulsions stabilized with 1.2% (*w*/*v*) emulsifiers stored at 25 °C for 30 d. Different lowercase letters (a–c) in subfigure D indicate significant differences (*p* < 0.05). Figure S3: Viscosity of PIDF suspensions at a particle concentration of 2% (*w*/*v*).
