# Supplementary material for: Emulsification Characteristics of Insoluble Dietary Fibers from Pomelo Peel: Effects of Acetylation, Enzymatic Hydrolysis, and Wet Ball Milling
Source: Foods. 2024 Feb 19;13(4):624. doi: 10.3390/foods13040624 (PMC10888415; doi:10.3390/foods13040624)
Supplement: Supplementary file 1 [file foods-13-00624-s001.zip › foods-2838884-supplementary.pdf]

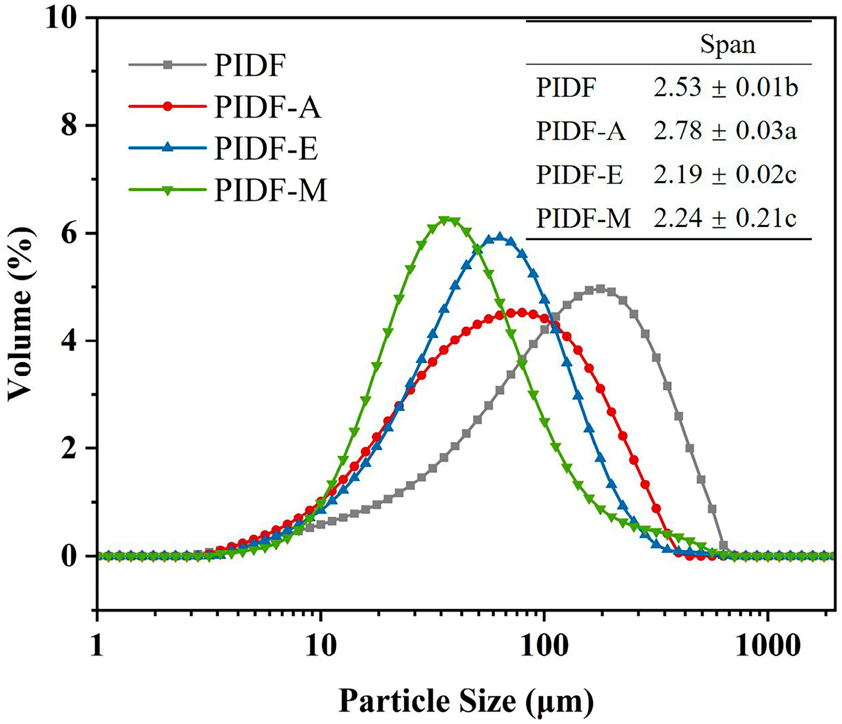

Figure S1: Particle size distribution of PIDF and the modified products. Different lowercase letters (a-c) indicate significant differences ($p < 0.05$). The value of the span parameter is calculated as $(D_{90} - D_{10}) / D_{50}$.

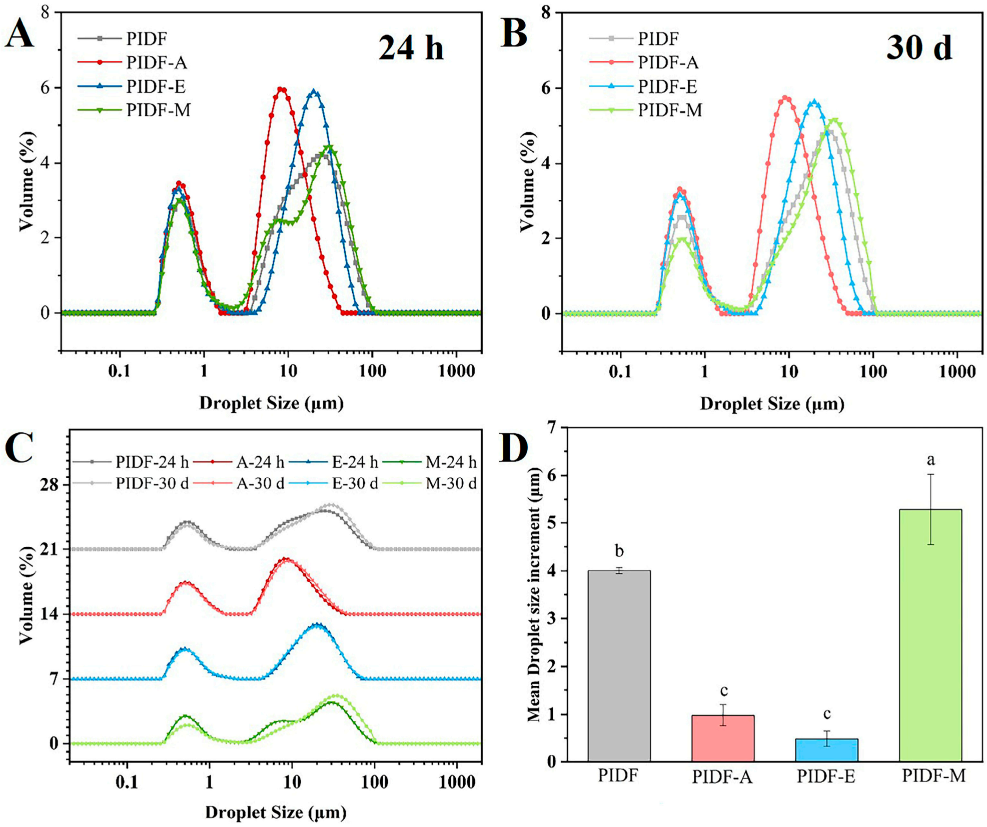

Figure S2: (A-C) Droplet size distributions of emulsions stabilized with 1.2% (w/v) emulsifiers, (D) Droplet size increment of emulsions stabilized with 1.2% (w/v) emulsifiers stored at 25°C for 30 d. Different lowercase letters (a-c) in subfigure D indicate significant differences ($p < 0.05$).

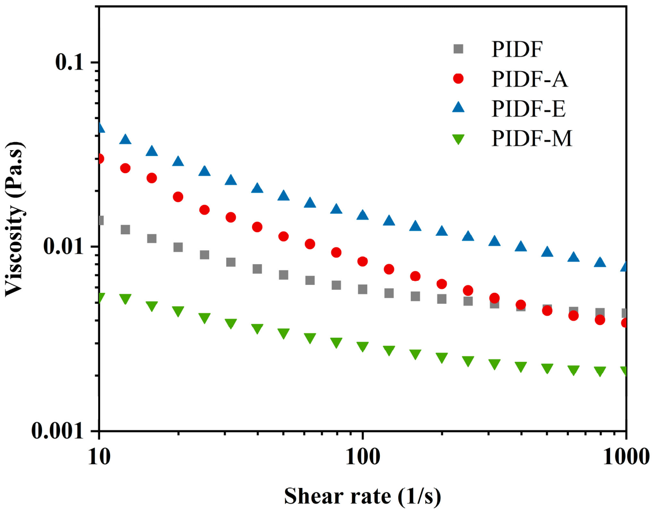

Figure S3: Viscosity of PIDF suspensions at a particle concentration of 2% (w/v). The apparent viscosities of PIDF suspensions (2% w/v, dissolved in distilled water) were determined on a rheometer (DHR2, TA Instruments, New Castle, DE, USA) as a function of shear rate (ranging from 10 to 1000 s^{-1}). The diameter of the parallel plate used was 40 mm, and the gap was set at 1000 μm. Among the modified PIDFs, PIDF-A and PIDF-E showed relatively higher viscosities, which may be related to their better hydrophilicity and larger specific surface area. Conversely, the tighter, more shallowly porous and less porous surface structure may hinder the aggregation and complexation of fibers in the aqueous phase, thus reducing the suspension viscosity.
